# Post-Operative Medium- and Long-Term Endocrine Outcomes in Patients with Non-Functioning Pituitary Adenomas—Machine Learning Analysis

**DOI:** 10.3390/cancers15102771

**Published:** 2023-05-16

**Authors:** Ziad Hussein, Robert W. Slack, Hani J. Marcus, Evangelos B. Mazomenos, Stephanie E. Baldeweg

**Affiliations:** 1 Department of Diabetes & Endocrinology, Sheffield Teaching Hospitals NHS Foundation Trust, Sheffield S10 2JF, UK; 2 Department of Diabetes & Endocrinology, University College London Hospital, London NW1 2BU, UK; s.baldeweg@ucl.ac.uk; 3 Centre for Obesity & Metabolism, Department of Experimental & Translational Medicine, Division of Medicine, University College London, London WC1N 3BG, UK; 4 Wellcome/EPSRC Centre for Interventional and Surgical Sciences, University College London, London W1W 7TY, UK; robert.slack.18@ucl.ac.uk (R.W.S.); h.marcus@ucl.ac.uk (H.J.M.); 5 Department of Medical Physics and Biomedical Engineering, University College London, London WC1E 6BT, UK; 6 Department of Neurosurgery, National Hospital for Neurology and Neurosurgery, London NW1 2BU, UK

**Keywords:** non-functioning pituitary adenoma, hypopituitarism, panhypopituitarism, machine learning, logistic regression, knn, svm, decision tree

## Abstract

**Simple Summary:**

Non-functioning pituitary adenomas (NFPAs) may present with hypopituitarism, and patients may develop or continue to exhibit hypopituitarism following surgery or radiotherapy to treat NFPAs. Panhypopituitarism is characterised as a deficiency in most or all pituitary hormones. Prediction of post-intervention hypopituitarism is currently limited, with no accurate models existing for medium- to long-term prognosis. The aim of this study was to develop machine learning (ML) models towards improving prediction of hypopituitarism up to 15 years following surgical intervention for NFPAs. Pre-operative hormone levels were shown to be the best predictor of panhypopituitarism up to 1 year post-operatively. Endocrine tests performed up to 1 year post-operatively were shown to support strong predictive models for assessing the probability of panhypopituitarism at 5 and 10 years post-operatively.

**Abstract:**

Post-operative endocrine outcomes in patients with non-functioning pituitary adenoma (NFPA) are variable. The aim of this study was to use machine learning (ML) models to better predict medium- and long-term post-operative hypopituitarism in patients with NFPAs. We included data from 383 patients who underwent surgery with or without radiotherapy for NFPAs, with a follow-up period between 6 months and 15 years. ML models, including k-nearest neighbour (KNN), support vector machine (SVM), and decision tree models, showed a superior ability to predict panhypopituitarism compared with non-parametric statistical modelling (mean accuracy: 0.89; mean AUC-ROC: 0.79), with SVM achieving the highest performance (mean accuracy: 0.94; mean AUC-ROC: 0.88). Pre-operative endocrine function was the strongest feature for predicting panhypopituitarism within 1 year post-operatively, while endocrine outcomes at 1 year post-operatively supported strong predictions of panhypopituitarism at 5 and 10 years post-operatively. Other features found to contribute to panhypopituitarism prediction were age, volume of tumour, and the use of radiotherapy. In conclusion, our study demonstrates that ML models show potential in predicting post-operative panhypopituitarism in the medium and long term in patients with NFPM. Future work will include incorporating additional, more granular data, including imaging and operative video data, across multiple centres.

## 1. Introduction

Non-functioning pituitary adenomas (NFPAs) are well-differentiated tumours arising from adenohypophysis of the pituitary gland, accounting for up to 54% of all pituitary adenomas [1]. Clinical presentation of these tumours varies, due to their lack of hormonal secretion and clinical syndromes, from being incidentally detected on radiological studies performed for other conditions to hypopituitarism and visual deficits secondary to mass effect on the pituitary gland and optic apparatus, respectively [2,3,4].

Hypopituitarism is defined as a deficiency of one or more pituitary hormones. A substantial number of patients with NFPAs develop a degree of hypopituitarism due to mechanical compression of the pituitary gland and reduction of pituitary portal circulation [1,5]. In addition, treatment with surgery with and without adjuvant radiotherapy may increase the risk of developing further pituitary impairment [4,6,7,8]. Currently, there are limited predictive models for post-operative endocrine outcomes in patients with NFPAs.

Previous studies have demonstrated the application of machine learning (ML) models in predicting individual pituitary hormone deficiencies immediately following transsphenoidal surgery for NFPAs [9]. The aim of this study was to extend this work by applying ML models to a larger cohort of patients with medium- and long-term follow-up.

## 2. Materials and Methods

Ethics approval to conduct this study was obtained from Westminster Research Ethics Committee on 7 April 2020. The Transparent Reporting of a multivariable prediction model for Individual Prognosis Or Diagnosis (TRIPOD) Statement was used in the preparation of this manuscript [10]. This study was a single-centre cohort study that included all patients who underwent surgical resection for NFPAs of more than 1 cm between 1987 and 2017 and had a follow-up duration of more than six months. The study was conducted at the National Hospital for Neurology and Neurosurgery based in London, United Kingdom. Surgical resection was performed mainly by three experienced neurosurgeons. A retrospective review of medical case notes was performed. Diagnosis of NFPAs was based on the absence of evidence of functioning tumours, e.g., hormone excess production and the expression of pituitary hormones in the resected tumours on immunohistochemistry. Data on patients’ demographics, endocrine assessment, treatment modalities, and tumour recurrence and regrowth were collected. A full list of fields and descriptions can be found in Appendix A.

Patients with severe growth hormone (GH) deficiency on dynamic testing (peak GH response < 3 µg/dL) or those on GH-replacement therapy were defined as GH-deficient [11]. Patients with combined thyroid stimulating hormone (TSH), adrenocorticotropic hormone (ACTH), and gonadotropins deficiencies without a dynamic test were also considered deficient in GH [12]. Patients with ACTH and TSH deficiencies were reported as deficient when patients received glucocorticoid and thyroxine therapy, respectively. In addition, patients who had a morning cortisol level of less than 100 nmol/L and those with a suboptimal response to dynamic testing were recorded as ACTH-deficient [13,14,15]. Patients with a low free T4 level with an inappropriately normal or low TSH level were recorded as TSH-deficient. Patients with primary hypothyroidism were not considered to have TSH impairment. Gonadotrophin deficiency was defined as follows: men were deficient if they were receiving testosterone therapy or if early morning testosterone level was low in the presence of inappropriately normal or low serum gonadotrophins [16]. Premenopausal women with amenorrhoea and inappropriately normal or low serum gonadotrophins were recorded as having hypogonadotrophic hypogonadism [17], while women who received oestrogen replacement in the form of hormone replacement therapy or oestrogen-containing contraceptive pills were also considered to be deficient in gonadotrophins. Postmenopausal women with non-elevated serum gonadotrophins were also documented as gonadotrophin deficient. Panhypopituitarism was considered when there was a deficiency of GH, gonadotrophins, ACTH, and TSH hormones. Patients on desmopressin replacement therapy were considered to have central diabetes insipidus (AVP deficiency).

Data were collected in Microsoft Excel before being imported into a series of Python scripts for analysis and visualisation (Python 3.9.7 64-bit|Windows 10). Key libraries used were pandas (v1.3.4), numpy (v1.20.3), sklearn (v1.1.2), and matplotlib (v3.4.3), and seaborn (v0.11.2) for visualisation. Conversion of text, numbers, and dates took place to ISO standard data types where possible.

For initial analysis, connections between surgeries and hormone deficiencies were investigated to draw broad connections between tumour behaviour following intervention and endocrine outcomes. Due to the established impact of radiotherapy on hormone deficiencies [18], an effect supported by this dataset, surgical interventions were initially investigated against the last valid endocrine test following the intervention, but before radiotherapy was administered, where applicable. For radiotherapy interventions, the last valid endocrine test following radiotherapy was used.

**Figure 1:**
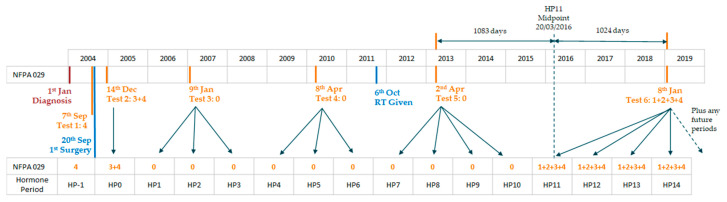
Example of endocrine timeline creation logic for Patient ID NFPA029, showing 1 year periods following the first surgery, taking endocrine status for each hormone period (HPx) from the closest test to the period mid-point (test results translate as: 0: no deficiency; 1: GH deficiency; 2: FSH/LH deficiency; 3: ACTH deficiency; 4: TSH deficiency).

In preparation for the application of ML algorithms, more sophisticated timelines were created for each patient for endocrine and radiological outcomes. These timelines were necessary in order to provide a chronology of endocrine and radiological findings granular enough to provide clinical insight and standardised for analysis across the full patient population. An example of the logic used is shown in Figure 1. Various timeline options were tested, with final results built around 6 month intervals, taken from the date of the first surgery. To reduce the risk of inaccurate classification for endocrine tests conducted on the same date as the surgical intervention, endocrine results were determined based upon the closest endocrine test to the mid-point of each period. Radiological outcomes were determined based on the closest radiological investigation to the end date of each period. For the purposes of visualising hormone deficiency populations, percentages were calculated over known endocrine profiles, with unknown results excluded.

For the application of ML algorithms, the endocrine and radiological outcome timelines were then filtered to focus the analysis. To maximise clinical relevance, hormone periods were restricted to the first 5 years following the first surgery. ML algorithms were applied to each subsequent radiological period, up to a maximum of 15 years. All hormone deficiencies were investigated, including the compound conditions for panhypopituitarism, and panhypopituitarism combined with AVP deficiency. Again, for clinical purposes, radiological outcomes were filtered to remove secondary outcomes (e.g., optic contact/compression, cavernous invasion, etc.). Finally, supplementary features within the source data then had the option of being added to the ML dataset, normalising where appropriate (age, tumour volume). Any features with only two possible values (e.g., sex), were reduced to a single Boolean feature. Features with multiple results (e.g., Second Radiology Scan), were converted to two features for each possible result (e.g., Second Scan Complete Resection: True, Second Scan Compete Resection: False). This ensured that potential missing data points (i.e., features not recorded), did not add bias in the ML models, allowing the developed algorithms to select for both the existence of a result and the absence of the same result in determining the best predictive model.

ML classification algorithms were applied with the aim of predicting post-operative panhypopituitarism up to 15 years following first surgery. Algorithms tested were simple logistic regression, k-nearest neighbour (KNN), support vector machine (SVM), and decision trees. These algorithms were selected due to their comparative strength in classification models over small datasets [19]. Due to the relatively small sample size, training data were prepared using 80% of patients, chosen at random. Model tests were then conducted against the remaining 20% forming the test dataset, and performance was captured against accuracy, F1, precision, recall, and area under the receiver operating characteristic curve (AUC-ROC). In each case, a confusion matrix was also generated. 

To account for potential model instability, each algorithm was run 20 times, varying the random seed each time, with all associated metrics assessed as the mean and standard deviation of the resulting output. Results of the algorithms were assessed against the best predictive capability for panhypopituitarism at 6 months, 1 year, 5 years and 10 years post-operatively. For the decision tree algorithm, results were captured for feature importance, using the GINI index, allowing extraction of the most influential features for each outcome period.

## 3. Results

### 3.1. Population Statistics

Simple population statistics for the patient cohort are shown in Table 1.

Pre-operatively, 235 patients (61%) had evidence of at least one pituitary deficiency. Anterior panhypopituitarism was more common in men (*p* = 0.001) and older patients (*p* = 0.005).

### 3.2. Hormone Deficiency by Intervention

**Figure 2:**
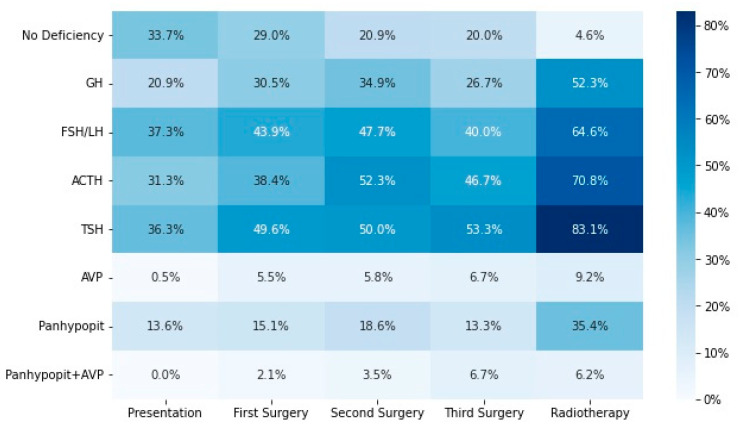
Heatmap showing percentages of patients with each hormone deficiency following each intervention. GH: growth hormone; FSH: follicle-stimulating hormone; LH: luteinising hormone; ACTH: adrenocorticotropic hormone; TSH: thyrotroph-stimulating hormone; AVP: arginine vasopressin; panhypopit: panhypopituitarism.

A heatmap of the percentage of patients exhibiting hormone deficiencies following each intervention is shown in Figure 2.

### 3.3. Hormone Deficiencies and Radiology Derived Timelines

**Figure 3:**
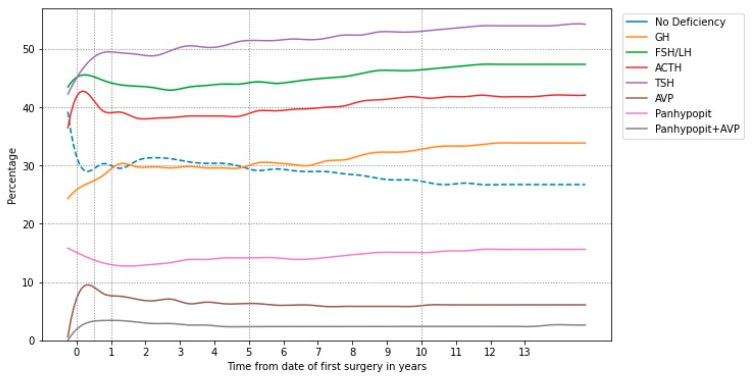
Hormone deficiency populations as a function of time following first surgery. GH: growth hormone; FSH: follicle-stimulating hormone; LH: luteinising hormone; ACTH: adrenocorticotropic hormone; TSH: thyrotroph-stimulating hormone; AVP: arginine vasopressin; panhypopit: panhypopituitarism.

Figure 3 shows the hormone deficiency rates as a function of time for the derived timelines, with specific rates shown at time of surgery and 6 months, 1 year, 5 years, and 10 years post-operatively, selected for clinical significance, included as Table A2 in Appendix A. Ten patients of those who underwent multiple surgeries without radiotherapy (number 46) had panhypopituitarism before secondary surgery; the rate rose to 12 patients at latest follow-up. In those treated with a combination of surgery and radiotherapy (number 65), 20 patients had panhypopituitarism after primary surgery; the incidence increased to 37 patients at latest clinical review.

### 3.4. Machine Learning for Determining Post-Operative Panhypopituitarism

Model accuracy and AUC-ROC comparison between the selected algorithms is shown in Table 2 as mean and standard deviation across all prediction periods. With minimal tuning, SVM and decision tree models showed superior accuracy and AUC-ROC scores. For ease of extraction of feature importance, the decision tree model was further analysed. For predictions up to 1 year post-operatively, pre-operative endocrine tests were included in the analysis for feature importance. For predictions at 5 and 10 years post-operatively, the most accurate early endocrine test period (up to one year following first surgery) was adopted for the analysis. The performance of the models when including the relative early endocrine test periods are shown in Table 3, with the feature importance for each future period shown in Table 4, in order of most to least important, excluding features with importance of less than 0.1, or where the standard deviation was equal to or greater than the feature importance. 

## 4. Discussion

There is no general agreement on predictors of hypopituitarism following therapy. Patients receiving surgery with and without radiotherapy for pituitary adenomas require long-term clinical follow-up and biochemical evaluation to identify pituitary impairment and instigate hormone replacement when appropriate [20]. In this study, we assessed several patients and tumour characteristics using ML models to predict the occurrence of pituitary insufficiency in a large cohort of patients with NFPAs following transsphenoidal surgery and post-operative radiotherapy. The principal finding of this study is that the likelihood of developing hypopituitarism was strongly influenced by the presence of pre-operative pituitary hypofunction.

Hypopituitarism refers to partial or complete loss of hormones produced by adenohypophysis or neurohypophysis that can be caused by several aetiologies causing damage in the hypothalamic–pituitary region. Pituitary adenomas and treatment with surgery and radiotherapy are considered the most common causes of hypopituitarism [6,20,21]. Clinical symptoms and presentation can be variable, from subclinical disease to life-threatening emergencies, dependent on the degree of hormone deficiency. At presentation, pituitary adenomas of more than 1 cm are frequently associated with pituitary hormone deficiency resulting from mass effect on the normal anterior pituitary and the pituitary stalk, preventing the stimulation of pituitary cells by the hypothalamus with a prevalence of up to 85% of cases [1]. The process of developing hormone deficiency can be insidious, slowly evolving, and delay in diagnosis is often common. Long-term hormone replacement is often required to improve quality of life and reduce morbidity and mortality, with lifelong follow-up recommended [22,23].

Transsphenoidal hypophysectomy remains the gold standard for treating large NFPAs [24,25], particularly when there is evidence of a pressure effect on the optic apparatus. The pituitary function can fluctuate following surgery and the risk of developing endocrine dysfunction post-operatively has been linked to many variables, including the size of the pituitary adenoma, the degree of surgical resection and manipulation, and dealing with recurrent disease [26]. The risk of deterioration in pituitary function ranges 0–36%, while recovery of deficient pituitary hormones varies 10–98% [6]. Hypothalamic pituitary dysfunction is one of the common late effects of pituitary radiotherapy. The occurrence of hypopituitarism following irradiation is progressive, and the severity of hormone deficits depends on many factors, including radiation dose, fraction size, the interval between fractions, and the duration of follow-up [8,27]. The exact mechanism is not very well understood; radiation-induced hypothalamic neural damage and secondary pituitary atrophy due to the loss of hypothalamic releasing and stimulating neurotransmitters are proposed to be contributing factors [28,29].

Panhypopituitarism, characterised here as simultaneous deficiencies across GH, gonadotrophins, ACTH and TSH, was present in 52 patients prior to surgery (13.6% of the total population; 15.8% of known endocrine profiles), and rose to 55 patients in the period immediately following surgery (14.4% of the population). This figure decreased marginally over the period 6–24 months after surgery, to a minimum of 49 patients, before steadily rising to 57 patients 10 years after initial surgery. 

ML models showed strong performance in predictions of panhypopituitarism up to 15 years following initial surgery, with the strongest-performing models being SVM (mean accuracy: 0.94; mean AUC-ROC: 0.88) and decision tree (mean accuracy: 0.92; mean AUC-ROC: 0.84). Both SVM and decision tree models exhibited stronger predictive capability than standard logistic regression (mean accuracy: 0.89; mean AUC-ROC: 0.79).

The decision tree model showed strong performance in predicting panhypopituitarism up to 1 year following initial surgery, based upon endocrine profiles taken pre-operatively. On analysis of feature importance within the model, only pre-operative presentation of panhypopituitarism showed a strong contribution (feature importance of pre-operative panhypopituitarism presence/absence: 0.40/0.32 and 0.39/0.33 when predicting panhypopituitarism at 6 months post-operative and 1 year post-operative, respectively), with weaker contributions from patient age and tumour volume. The same model showed strong performance in the prediction of panhypopituitarism at 5 and 10 years following initial surgery, based upon endocrine profiles taken 1 year post-operatively. For these periods, only the prior presentation of panhypopituitarism (with or without AVP deficiency) contributed strongly to the model (feature importance of 1 year post-operative panhypopituitarism presence: 0.76 and 0.71 when predicting panhypopituitarism at 5 years post-operative and 10 years post-operative, respectively), with weak contribution from follow-up intervention with radiotherapy.

A recent study by Fang et al. [9], the only study that assessed pituitary outcomes using ML models after surgery, reported that pre-operative adenohypophysial endocrine impairment was associated with a higher risk of developing hormone deficiency post-operatively. Our study findings are in line with Fang et al.’s conclusion and extend ML experience of anticipating hypopituitarism in a large cohort of patients, of both genders, with NFPAs and a long follow-up duration. A limitation of the study concerns the inconsistent timing of endocrine assessments across the cohort and the resultant need to create standardised timelines algorithmically. Furthermore, the limitation of retrospective data collection applies.

## 5. Conclusions

ML models have been shown to provide stronger predictive performance than standard statistical methods when assessing patients’ prognosis for hypopituitarism following surgical intervention to treat NFPAs. Investigation of a feature-rich patient dataset (383 cases), including population demographics, endocrine assessment, treatment modalities and results, and radiology scan results, indicated prior presentation of panhypopituitarism as the strongest predictor of post-operative panhypopituitarism. Minor influence was observed from the patient’s age, tumour volume, and post-operative treatment with radiotherapy. ML models can be employed to predict endocrine outcomes in pituitary adenomas as demonstrated in this study. Further research is needed to develop accurate and reliable ML algorithms to aid in identifying prognostic markers in pituitary adenomas. Patients with pre-operative pituitary impairment in NFPAs are at a higher risk of persistent hypopituitarism following surgery or in combination with radiotherapy. This cohort of patients requires long-term endocrine monitoring to optimise hormone replacement and overall outcome.

## Figures and Tables

**Table 1:** Patient cohort summary.

Measure	Value
Total patients	383
Mean age	56.8
Std age (years)	13.5
Sex ratio (male:female)	67:33
% of patients receiving one or more surgeries	100
% of patients receiving two or more surgeries	22
% of patients receiving three surgeries	4
% of patients receiving radiotherapy	17

**Table 2:** Comparison of panhypopituitarism model performance, taken as the mean and standard deviation of accuracy and AUC-ROC across early hormone deficiency periods, for all future periods being modelled.

	Accuracy		AUC-ROC	
	Mean	Std	Mean	Std
Logistic regression	0.89	0.03	0.79	0.07
KNN	0.87	0.04	0.61	0.06
SVM	0.94	0.03	0.88	0.06
Decision tree	0.92	0.03	0.84	0.06

**Table 3:** Best theoretical early endocrine test period for determining panhypopituitarism as an outcome, as measured using model accuracy and AUC-ROC for decision tree algorithm.

Time from First Surgery	Early Endocrine Test Period	Mean Accuracy	Std Accuracy	Mean AUC-ROC	Std AUC-ROC
6 Months	Pre-operative	0.89	0.04	0.77	0.09
1 Year	Pre-operative	0.88	0.04	0.77	0.09
5 Years	1 year post-operative	0.97	0.03	0.95	0.03
10 Years	1 year post-operative	0.94	0.02	0.92	0.06

**Table 4:** Feature importance in predicting panhypopituitarism following first surgery. AVP: arginine vasopressin; panhypop: panhypopituitarism.

Prediction Period	Feature	Mean Importance	Std Importance
6-months post-operative	Panhypop @ Pre-operative: True	0.40	0.38
	Panhypop @ Pre-operative: False	0.32	0.37
	Age	0.07	0.02
	Tumour Volume (cc)	0.04	0.02
1 year post-operative	Panhypop @ Pre-operative: True	0.39	0.36
	Panhypop @ Pre-operative: False	0.33	0.37
	Age	0.08	0.04
	Tumour Volume (cc)	0.07	0.03
5 years post-operative	Panhypop @ 1 year Post-operative: True	0.76	0.03
	Panhypop + AVP @ 1 year Post-operative: True	0.18	0.03
	Radiotherapy already given: True	0.02	0.01
10 years post-operative	Panhypop @ 1 year Post-operative: True	0.71	0.04
	Panhypop + AVP @ 1 year Post-operative: True	0.19	0.06
	Radiotherapy already given: True	0.02	0.01

## Data Availability

The data presented in this study will be made publicly available, upon the paper’s acceptance, in the University College London institutional research repository (https://rdr.ucl.ac.uk/ (accessed on 15 May 2023)).

## Appendix A

**Table A1:** Complete list of data fields and their descriptions.

Fieldname	Description
date_of_birth	Patient’s date of birth
age	Approximate decimal age of the patient in years on the date of diagnosis
gender	Biological sex of the patient
clinical_presentation	Clinical presentation of the patient. Conditions include: Incidental; Headache; Visual symptoms; Hypopituitarism; Apoplexy; Others; Unknown; Hyponatraemia; Hydrocephalus; facial numbness; cranial n palsy; Hyposmia.
date_of_diagnosis	Date of Diagnosis
date_first_surgery	Date of each of the patient’s surgeries, where applicable
date_second_surgery	
date_third_surgery	
approach_first_surgery	Surgical approach taken for each surgery: transsphenoidal (TSS) or transcranial (TCS)
approach_second_surgery	
approach_third_surgery	
surgeon_first_surgery	Lead neurosurgeon on each surgery
surgeon_second_surgery	
surgeon_third_surgery	
preop_vision	Measure of the patient’s vision prior to first surgery. Possible options: Normal; Visual Loss; Unknown
postop_vision	Measure of the patien’ts vision after first surgery. Possible options: Normal; Improved; No change; Unknown; Deteriorated
date_radiotherapy_offered	Date on which radiotherapy was offered to the patient, where applicable (Not used in analysis)
date_radiotherapy_given	Date on which radiotherapy treatment commenced, where applicable
radiotherapy_type	Type of radiotherapy administered, where applicable. Possible options: Conventional radiotherapy; Gammaknife; Stereotactic radiotherapy
date_ of_test_first	Date of each endocrine test
date_ of_test_second	
date_ of_test_third	
date_ of_test_fourth	
date_ of_test_fiveth	
date_ of_test_sixth	
hormone_deficit_first	Results of each endocrine test, categorised as described in Section 2: Materials and Methods
hormone_deficit_second	
hormone _deficit_third	
hormone_deficit_fourth	
hormone_deficit__fiveth	
hormone_deficit_sixth	
pathology_outcome_post_first_surgery	Endocrine profile taken immediately after surgery. Possible outcomes: FSH; LH; Not applicable; Null cell; ACTH.
pathology_outcome_post_second_surgery	
pathology_outcome_post_third_surgery	
Ki 67_first_surgery	Ki67 mitotic proliferation measure, taken following each surgery. Possible outcomes: <1%; 1–3%; 3–5%; >5%.
Ki 67_second_surgery	
Ki 67_third_surgery	
frequent_mitosis_first_surgery	Frequent mitosis proliferation measure, taken following each surgery. Possible outcomes: Normal; Increased; Unknown
frequent_mitosis_second_surgery	
frequent_mitosis_third_surgery	
date_of_scan_first	Date of each radiology scan administered. Typically, the first scan occurred pre-operatively and second scan occurred post initial surgery, with subsequent scans taken over follow-up periods.
date_of_scan_second	
date_of_scan_third	
date_of_scan_fourth	
date_of_scan_fiveth	
date_of_scan_Sixth	
date_of_scan_seventh	
date_of_scan_eighth	
Scan_first	Results of the first radiology scan. Possible outcomes: Macroadenoma; Optic n compression; Cavernous sinus invasion; Sphenoid sinus invasion; Clivus invsion; Ethmoid invsion; Hydrocephalus; Unknown; Nasopharynx; Ventricle
Scan_second	Results of the second radiology scan. Possible outcomes: Complete resection; Residual tumour (RT); RT + Optin n compression; RT + Cavernous invasion; RT + Sphenoid sinus; Unknown; RT + Op n + CS invasion; Rt + Clivus.
Scan_third	Results of follow-up radiology scans. Possible outcomes: No recurrence; Residual tumour Stable; increase in size; optic contact/compression; Cavernous invasion; Sphenoid; Clivus/skull base; Recurrence; Reduction in size; Residual tumour.
Scan_fourth	
Scan_fiveth	
Scan_Sixth	
Scan_seventh	
Scan_eighth	
date_last_seen	Date of last clinical visit
cc	Estimated tumour volume (cc)
transverse	Transverse tumour length (mm)
ap	Anterior-posterior tumour length (mm)

**Table A2:** Population percentages exhibiting hormone deficiencies over clinically relevant periods, extracted from derived patient timelines. GH: Growth Hormone; FSH: Follicle Stimulating Hormone; LH: Luteinising Hormone; ACTH: Adrenocorticotropic Hormone; TSH: Thyrotroph Stimulating Hormone; AVP: Arginine Vasopressin.

	Time Relative to First Surgery					
	Pre-Operative	0–6 Months	6–12 Months	1–1.5 Years	5 Years	10 Years
No Deficiency	39.2%	29.1%	30.3%	29.5%	29.1%	27.0%
GH	24.3%	26.7%	28.2%	30.3%	30.4%	33.1%
FSH/LH	43.5%	45.5%	44.6%	43.9%	44.4%	46.6%
ACTH	36.5%	42.7%	39.4%	39.2%	39.4%	41.5%
TSH	42.2%	47.1%	49.3%	49.3%	51.4%	53.2%
AVP	0.6%	9.4%	8.1%	7.6%	6.3%	6.1%
Panhypopituitarism	15.8%	14.4%	13.3%	12.8%	14.2%	15.1%
Panhypopituitarism + AVP	0.0%	2.9%	3.4%	3.4%	2.4%	2.4%
